# Adaptation and exogenous selection in a *Picea glauca* × *Picea engelmannii* hybrid zone: implications for forest management under climate change

**DOI:** 10.1111/nph.12540

**Published:** 2013-10-08

**Authors:** Amanda R De La Torre, Tongli Wang, Barry Jaquish, Sally N Aitken

**Affiliations:** 1 Centre for Forest Conservation Genetics, Department of Forest and Conservation Sciences, University of British Columbia3041–2424 Main Mall, Vancouver, BC, V6T1Z4, Canada; 2 Department of Ecology and Environmental Sciences, Umeå UniversityLinneaus väg 6, SE-901 87, Umeå, Sweden; 3 Kalamalka Forestry Centre, Tree Improvement Branch, BC Ministry of Forests, Lands & Natural Resource Operations3401 Reservoir Rd, Vernon, BC, V1B2C7, Canada

**Keywords:** adaptive introgression, bounded hybrid superiority, breeding, climate change, exogenous selection

## Abstract

- The nature of selection responsible for the maintenance of the economically and ecologically important *Picea glauca × Picea engelmannii* hybrid zone was investigated. Genomic, phenotypic and climatic data were used to test assumptions of hybrid zone maintenance and to model future scenarios under climate change.
- Genome-wide estimates of admixture based on a panel of 86 candidate gene single nucleotide polymorphisms were combined with long-term quantitative data on growth and survival (over 20 yr), as well as one-time assessments of bud burst and bud set phenology, and cold hardiness traits. A total of 15 498 individuals were phenotyped for growth and survival.
- Our results suggest that the *P. glauca* × *P. engelmannii* hybrid zone is maintained by local adaptation to growing season length and snowpack (exogenous selection). Hybrids appeared to be fitter than pure species in intermediate environments, which fits expectations of the bounded hybrid superiority model of hybrid zone maintenance. Adaptive introgression from parental species has probably contributed to increased hybrid fitness in intermediate habitats.
- While *P. engelmannii* ancestry is higher than *P. glauca* ancestry in hybrid populations, on average, selective breeding in managed hybrid populations is shifting genomic composition towards *P. glauca*, potentially pre-adapting managed populations to warmer climates.

## Introduction

The role of hybridization in adaptive evolution has been a contentious issue in evolutionary biology. Some researchers have argued that hybridization is a potent evolutionary force that facilitates adaptive evolution and can lead to new species (Anderson, 1949; Arnold, 1997; Abbott *et al*., 2013). According to this perspective, new gene combinations resulting from hybridization may promote the development of adaptations to novel or changing environments (Rieseberg *et al*., 2003). By contrast, natural hybridization between divergent populations has been considered an evolutionary dead end because it can lead to unfit or unviable hybrids (Barton & Hewitt, 1985). With climates changing rapidly as a result of anthropogenic warming, the effects of hybridization and introgression on the rate of adaptation are important for both natural and managed populations, particularly for species with long generation lengths such as trees (Aitken *et al*., 2008).

Although definitive evidence of whether hybridization more often promotes or hinders adaptation is lacking, recent studies have supported the former for many plant species (Choler *et al*., 2004; Campbell & Waser, 2007; Stift *et al*., 2008; De Carvalho *et al*., 2010; Thomasset *et al*., 2011). In sunflower (*Helianthus* spp.), new gene combinations generated by introgression have contributed to ecological divergence (Rieseberg *et al*., 2003), and adaptation in several abiotic tolerance traits (Whitney *et al*., 2010). Adaptive introgression has increased flooding tolerance in *Iris* (Martin *et al*., 2005, 2006) and *Rorippa* (Stift *et al*., 2008), drought tolerance in *Pinus* (Ma *et al*., 2010) and light tolerance in *Silene* (Goulson, 2009).

The maintenance of hybrid zones has been the subject of considerable debate, mainly because of different points of view about the relative powers of natural selection as a divergent force and gene flow as a homogenizing force (Harrison, 1993; Barton, 2001). Environment-independent models describe hybrid zones maintained by a balance between dispersal and selection against hybrids, with selection being independent of the environment (Mayr, 1942; Barton, 1979). The *tension zone* model assumes that hybrids are less fit than their parents regardless of location (endogenous selection; Gay *et al*., 2008; Carling & Zuckerberg, 2011). Hybrid inferiority is thought to result from the break-up of epistatic co-adapted gene complexes that affect fitness traits (Barton & Hewitt, 1985; Hewitt, 1988). Environment-dependent models involve genotype-by-environment interactions, where hybrid zones are maintained through selection gradients resulting from environmental heterogeneity (Endler, 1973, 1977; Slatkin, 1973; Harrison, 1986). In these models, hybrid fitness varies with the environment (exogenous selection). The *bounded hybrid superiority* model (Moore, 1977) is an important environment-dependent model, which postulates that hybrid individuals are fitter than either parental species in environments that are intermediate to parental habitats, but are less fit than parental species in their respective native habitats (Wang *et al*., 1997; Milne *et al*., 2003; Miglia *et al*., 2005; Goulson, 2009).

*Picea glauca* (Moench) Voss (white spruce) and *Picea engelmannii* Parry ex Engelm. (Engelmann spruce) are closely related, wind-dispersed, long-lived tree species that hybridize extensively in areas where their ranges overlap, mostly in British Columbia and the western part of Alberta, Canada. *Picea glauca* and *P. engelmannii* inhabit different ecological niches separated primarily by elevation. *Picea engelmannii* is a subalpine species that has relatively low tolerance of high temperatures and drought, but can withstand short growing seasons and high snowfall (Alexander & Shepperd, 1990). *Picea glauca* is primarily a boreal species that grows under highly variable conditions and can tolerate both low winter temperatures and summer drought, but is restricted to low elevations. Hybrids occupy ecological and elevational niches intermediate to those of the parental species. Hybridization followed by backcrossing has produced hybrids with intermediate characteristics in morphology and phenotypic traits, and clinal variation along elevational gradients (Roche, 1969; Daubenmire, 1974; Rajora & Dancik, 2000; Ledig *et al*., 2006). Neutral molecular marker studies have found clines in admixture along elevational and latitudinal gradients, with hybrids on average having a higher contribution from *P. engelmannii* than from *P. glauca* (A. R. De La Torre & S. Aitken, unpublished data). Although it has been suggested that hybrids are fitter than parentals in intermediate environments (Roche, 1969; Daubenmire, 1974; Xie *et al*., 1998; Ledig *et al*., 2006), this has never been tested.

*Picea glauca* and *P. engelmannii* are economically important tree species in Canada. The complex of these two species and their hybrids (collectively managed as ‘interior spruce’) represents the second most planted tree in British Columbia. Tens of millions of trees are planted annually for reforestation in the province, the majority of which are products of a large breeding programme that was initiated in the early 1960s. With British Columbia getting warmer and in some areas drier as a result of climate change (Mbogga *et al*., 2009), there is a need to develop climate-informed strategies to manage the spruce genetic resource. In order to develop these management strategies, it is important to have a thorough understanding of the hybrid zone complex and the effects of artificial selection on the genomic architecture of this zone.

Our study is the first to assess the evolutionary dynamics responsible for the maintenance of the *P. glauca × P. engelmannii* hybrid zone by combining genome-wide estimates of admixture, quantitative data from long-term common garden experiments, and environmental data. By doing so, we expect to contribute valuable information for current and future management of this species complex. Our objectives are: to test the bounded hybrid superiority model of hybrid zone maintenance by classifying individuals using a single nucleotide polymorphism (SNP)-based hybrid index, comparing the fitnesses of hybrids and parental species in extensive common garden experiments, and elucidating the effects of climate on the distribution of genetic variation within the hybrid zone; and to assess the effects of ongoing selective breeding and projected climate change on genetic composition within the hybrid zone.

## Materials and Methods

### Genetic materials

**Figure 1:**
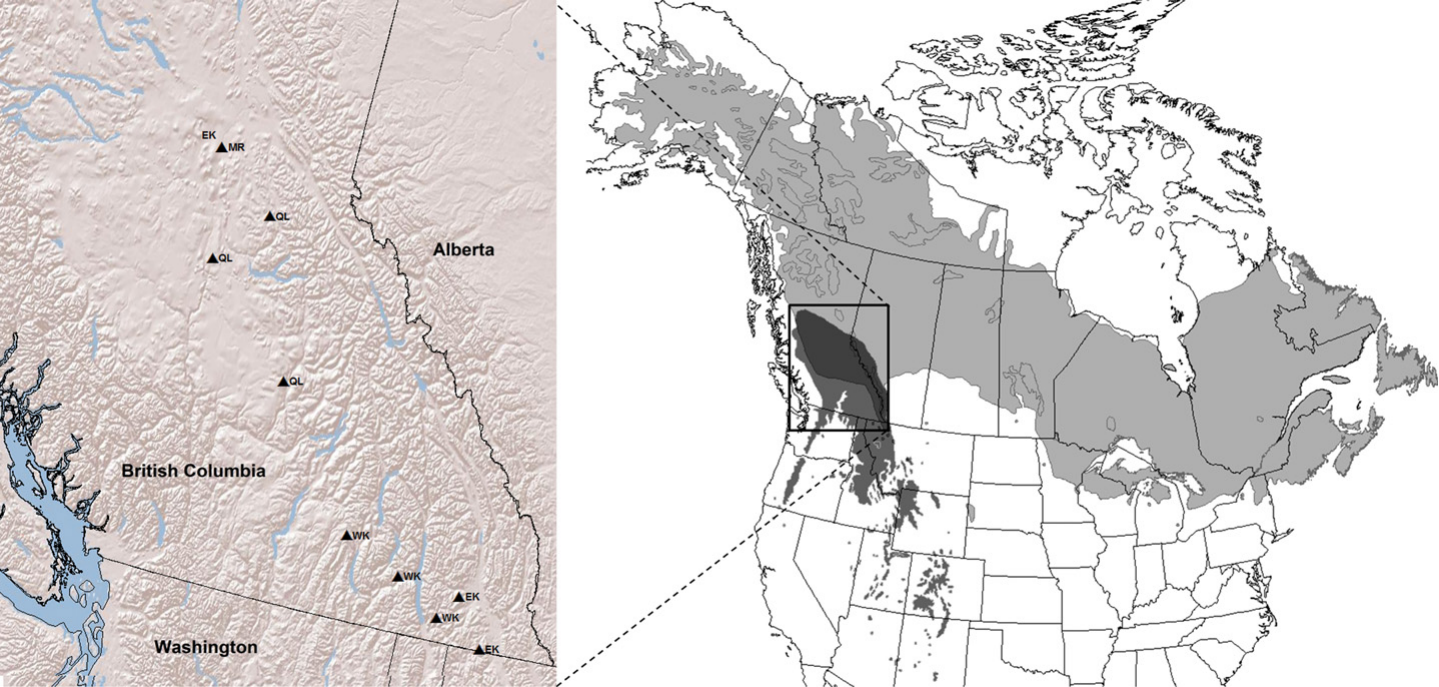
Geographical location of *Picea glauca* × *Picea engelmannii* common garden experiments in each of the four geographical regions (seed planning zones (SPZ)) studied in British Columbia, Canada. EK, East Kootenay; WK, West Kootenay; MR, Mouth Robson; QL, Quesnel Lakes.

Individuals genotyped and phenotyped in this study were collected and established by the interior spruce breeding programme of the British Columbia Ministry of Forests, Lands and Natural Resources Operations. Seed planning zones (SPZs) are local geographical units for genetic management based on ecological and quantitative trait variation. Trees in four SPZs were sampled, genotyped and phenotyped: West Kootenay, East Kootenay, Quesnel Lakes, and Mount Robson (Table1; Fig.1). Seed parents of these progeny originated from known locations within natural stands, and had a diffuse distribution across SPZs in terms of latitude, longitude, elevation and climate. Within each SPZ, a few hundred open-pollinated families are represented in several progeny tests. We selected a subset of 200 families (50 in each SPZ) to obtain a relatively uniform distribution of seed parent origins along elevational gradients within zones. Newly flushed needles were sampled from up to four randomly selected progeny from each family in a single progeny test for genotyping, with a total of 745 individuals sampled. In addition, 22 putatively pure *P. glauca* individuals from Fort Nelson (FN) and 40 putatively pure *P. engelmannii* from southwestern USA (populations E1, E2, and E3) were obtained for genotyping from grafts of mature trees sampled from natural populations and archived in clone banks. Trees from a low-elevation test site (*P. glauca* habitat) in Fort Nelson were also phenotyped.

**Table 1:** Geographical coordinates and climatic variables for *Picea glauca* × *Picea engelmannii* common garden experiments studied

SPZ	Site	Reps	*N*	Elevation (m)	Latitude (°)	Longitude (°)	MAT (°C)	MAP (mm)	PAS (mm)	DD < 0	AH:M	Hab.
EK	Bloom Ck	2	374	1676	49.0	115.4	2.1	1081	627	1111	11.2	hyb
	Perry Ck	3	542	1463	49.5	116.0	2.1	703	372	1138	17.2	hyb
	Red Rock	9	979	762	53.7	122.7	3.5	599	217	1008	22.5	hyb
WK	Hall Ck	10	1050	1200	49.2	116.3	3.8	820	393	895	16.8	hyb
	Duhamel	10	1099	1475	49.6	117.2	2.1	1103	608	1082	11	hyb
	Cortiana	10	1207	1830	49.9	118.3	1.3	1100	562	1171	10.3	*P. eng*
QL	Little Benson	8	1744	960	52.5	122.3	3	590	212	996	22	hyb
	Camp Ck	8	1727	1080	51.4	120.3	3.3	638	260	936	20.8	hyb
	Ketcham Ck	8	1760	1380	53.1	121.4	1.4	864	382	1141	13.2	hyb
MR	Red Rock	8	1600	762	53.7	122.7	3.5	599	217	1008	22.5	hyb
FN	Fort Nelson	8	3416	600	58.7	123.7	−0.7	571	158	2113	16.3	*P. glau*

The number of individuals (*N*) represents a subset of the total number of individuals planted at each site.

EK, East Kootenay; WK, West Kootenay; QL, Quesnel Lakes; FN, Fort Nelson; MR, Mount Robson (not included in fitness analysis); SPZ, seed planning zone; Reps, number of replicates; *N*, number of individuals; MAT, mean annual temperature; MAP, mean annual precipitation; PAS, precipitation as snow; DD < 0, degree-days below 0°C; AH: M, annual heat: moisture index (MAT + 10)/(MAP/1000); Hab, habitat; hyb, hybrid; *P. eng*,* P. engelmannii*;* P. glau*,* P. glauca*.

### DNA extraction and genotyping

Needles were stored at −80°C before DNA isolation. Each sample was isolated using a modified cetyltrimethyl ammonium bromide (CTAB) protocol (Doyle & Doyle, 1987). The DNA quality and concentration of each sample was assessed qualitatively using 0.8% agarose gels, and quantitatively using Nanodrop 2000C Spectrophotometer readings (Thermo Fisher Scientific Inc., Waltham, MA, USA). All DNA samples were SNP genotyped at the Genome Quebec/McGill Innovation Centre (Quebec, Canada) using an Illumina bead array chip (Illumina Inc., San Diego, CA, USA) in conjunction with the GoldenGate allele-specific assay in a 96-well, 768-SNP format (Fan *et al*., 2003; Shen *et al*., 2005). Samples from allopatric pure species populations and from hybrid populations were assayed in two different SNP arrays. In the first SNP array, allopatric *P. glauca* and *P. engelmannii* samples were tested using 1536 SNPs from a large panel of genes putatively involved in cold hardiness (Holliday *et al*., 2008, 2010), insect herbivory resistance (Porth *et al*., 2011) and growth and bud set timing (Namroud *et al.,* 2008; Pavy *et al*., 2008; Pelgas *et al*., 2011). SNP selection and quality criteria are described in detail in A. R. De La Torre *et al*., submitted. In the second SNP array (96-well, 386-SNP format), SNPs selected from the first array (384) were used to genotype 745 samples from the hybrid zone. Of 384 SNPs selected, 86 were successfully genotyped in both pure species and hybrid samples and met both genotyping quality and data normalization criteria (De La Torre *et al*., 2013).

### Hybrid index and hybrid classes

The hybrid index was calculated using 86 SNP loci with the introgress 1.1 (Gompert & Buerkle, 2010) package in R 2.13.1 (R Development Core Team, 2011). To estimate the hybrid index, the Fort Nelson (FN) population was used as a parental *P*. *glauca* population and E1, E2 and E3 were used as parental *P. engelmannii* populations. Individuals were divided into four genotypic categories according to their hybrid index values as follows: 0–0.199, *P. glauca*; 0.2–0.499, *P. glauca* like-hybrids; 0.5–0.799, *P. engelmannii* like-hybrids; 0.8–1, *P. engelmannii*.

Genotypic classes were also estimated using the program NewHybrids 1.1 (Anderson & Thompson, 2002). NewHybrids uses the Markov chain Monte Carlo (MCMC) simulation for computing the posterior distribution of hybrid categories into which individual samples fall. Runs were tested using 20 000 sweeps. Individuals representing pure species were identified *a priori* and originated from populations outside of the hybrid zone. The default genotype frequency classes were used for the calculations. Following the NewHybrids assignment, individuals were divided into four groups: *P. glauca*,* P. engelmannii*, F1 hybrids, and Fn hybrids (advanced generation hybrids, including backcrosses and F2).

The phenotyped progeny tests described above (Genetic materials section) lacked test sites in *P. glauca* habitats. To address this shortcoming for our phenotypic analysis, we included data from the low-elevation test site at Fort Nelson. As we lacked genotypic data for these individuals, we assigned individuals to genotypic classes based on our earlier analysis of the hybrid index along elevational and latitudinal clines (A. R. De La Torre *et al*., unpublished data) as follows: individuals from below 600 m or originating from northern British Columbia, Alberta or Eastern Canada were classified as *P. glauca* (213 individuals); individuals from above 1800 m (mainly southwestern USA) were classified as *P. engelmannii* (224 individuals); all other individuals were classified as hybrids (2680 individuals).

### Phenotypic data

Phenotypic data were available for three progeny test sites in each of the West Kootenay, East Kootenay and Quesnel SPZs, and for one test site for Mount Robson (Table1). Each site was planted as a randomized complete block experiment with two to ten blocks. Tree height was measured and survival recorded at each site in most or all SPZs at ages 3, 6, 10 and 20 yr. Phenotypic data were also available for the Fort Nelson test site for growth traits (height and survival at age 6 yr). A total of 15 498 individuals were phenotyped and analysed in the five SPZs. The sites between 762 and 1676 m elevation were classified as hybrid sites, the site above 1800 m in the West Kootenay SPZ was classified as a *P. engelmannii* site, and the Fort Nelson site at 600 m elevation was classified as a *P. glauca* site (Table1).

Timing of bud burst was assessed on a subset of trees in the common garden experiments in spring of 2009 at the time of sampling foliage for DNA extraction and genotyping. Bud break was recorded for the terminal bud of lateral branches using a system that classifies bud burst stages from one to eight according to the amount of bud swelling, colour and elongation (Alfaro *et al*., 2000). Bud set data were not recorded in these common gardens, but data on the timing of bud set were available for 2-yr-old seedlings from a subset of the same East Kootenay open-pollinated families from an earlier seedling common garden (O′Neill & Aitken, 2004).

Needles from the current year′s growth from the same families in East Kootenay, Mount Robson, Quesnel, and West Kootenay were sampled in late August 2010 to artificially test fall cold hardiness. A total of 931 individuals from 184 families (four to eight progeny per family) were tested. Cold hardiness was measured using electrolytic leakage as a proxy for cell death. Details of the freeze-testing methodology are described by Hannerz *et al*. (1999). Briefly, freeze-testing temperatures were chosen *a priori* based on the results of a cold-hardiness pre-test using five temperatures (−10, −20, −30, −40 and −50°C) and 15 interior spruce individuals. Five-millimetre needle segments from each tree sampled were tested at −25, −35 and −45°C in a temperature chamber, with an unfrozen sample for a control. Samples were frozen in 0.2 ml of deionized water with a small trace amount of AgI to nucleate freezing, and an additional 2 ml of deionized water was added following freezing. Electrolytic leakage was measured for each sample after freezing, and again after being heat killed. An index of injury (*I*_*t*_) for each of the frozen samples was calculated as the percentage of injury that occurred, ranging from zero (control) to 100 (heat-killed), as described by Flint *et al*. (1967):

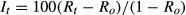
(*R*_*t*_ = *L*_*t*_/*L*_k_; *R*_o_ = *L*_o_/*L*_d_; *L*_*t*_, the conductance of leachate from the sample frozen at temperature *t*;* L*_k_, the conductance of leachate from the sample frozen at temperature *t* and heat-killed; *L*_o_, the control (unfrozen sample); *L*_d_, the conductance of the leachate from the heat-killed control (unfrozen sample; Hannerz *et al*., 1999).

### Fitness analysis

Height, survival, cold hardiness, bud burst, and bud set were used as fitness proxies to compare the performance of pure species and hybrids at different ages and between different environments within SPZs. Differences between pure species and hybrids for height at different ages were assessed by ANOVA using the following linear model:

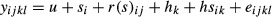
(*y*_*ijkl*_, the phenotype of individual *l* in hybrid class *k* in replication *j* of site *i*;* s*_*i*_, the effect of site *I*;* r*(*s*)_*ij*_, the effect of replication *j* within site *I*;* h*_*k*_, the effect of hybrid class *k*;* hs*_*ik*_, the interaction between hybrid class *k* and site *I*;* e*_*ijkl*_, the error.) A simplified regression model was used for bud burst, bud set, and cold hardiness traits.

Least square means with corresponding *F* tests and *P* values were estimated for each genotypic class in each SPZ. Variance components were estimated using the restricted maximum likelihood method with type III sum of squares. For bud burst, bud set and cold hardiness traits, population means were used in regression analyses. Bonferroni multiple comparison tests were used to detect differences between means of genotypic classes. Survival data were analysed using binomial tests for contingency tables. All procedures were conducted using either sas enterprise guide 4.2 or sas 9.2 (SAS Institute Inc, 1989) and graphed with R 2.13.1 (R Development Core Team, 2011).

### Climate analysis

ClimateWNA (Wang *et al*., 2012) was used to generate climate data for each sampling site and for a spatial grid (1 × 1 km) of the study areas. ClimateWNA downscales PRISM (parameter-elevation regressions on independent slopes model) (Daly *et al*., 2002) monthly data (2.5 × 2.5 arcmin) for the reference period (1961–1990), and calculates a large number of additional climate variables for specific locations based on latitude, longitude and elevation for western North America. Twenty annual climate variables were used to test for relationships between hybrid index, geography and climate (Supporting Information Table S7). Two climate data sets were generated for the reference period 1961–1990 (‘1970s’) and the future period 2041–2070 (‘2050s’).

To relate the hybrid index to climate, all climate variables, their combinations and transformations (quadratic and inverse forms) were screened using univariate and stepwise multivariate regression procedures in sas (SAS Institute Inc., Cary, NC, USA). In the multivariate analysis, samples from different SPZs were pooled. Hybrid indices were predicted based on the climate–hybrid index relationships developed in this study. Predicted hybrid indices were then mapped using ArcGIS (v. 10) for the two geographical regions where sampling sites were located (Fig. 5). To demonstrate the impact of climate change on the climatic niche for the hybrids, hybrid index was projected for a middle-of-the-road future climate scenario for the 2050s using the CGCM3 A2 run4.

### Effects of artificial selection on hybrid index

In order to assess the effect of artificial selection on the hybrid index, parental breeding values (Falconer & Mackay, 1996) for height growth were correlated with mean hybrid index estimates based on genotypic data for their open-pollinated progeny for each SPZ. The Best Linear Prediction method (White & Hodge, 1989) was used to calculate the breeding values of each parent in each SPZ, according to:

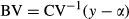
(**C**, a vector of genetic covariance between the observed half-sib family means and the breeding value being predicted; **V**, an s × s matrix of the phenotypic variances and covariances of family means; *y*, the progeny mean of the parent (family mean); α, the progeny mean of all parents (population mean or overall family mean).

## Results

### Hybrid index

The distribution of genotypic classes was asymmetrical, weighted towards Engelmann spruce, with the hybrid genotypic class between 0.5 and 0.799 accounting for nearly 60% of all individuals sampled. This asymmetry towards Engelmann spruce was observed in previous studies in the same hybrid complex (Haselhorst & Buerkle, 2013; A. R. De La Torre *et al*., unpublished data). Individuals with genotypic classes between 0 and 0.199 (putatively pure *P. glauca*) accounted for just 4% of the individuals.

### Fitness analysis

**Figure 2:**
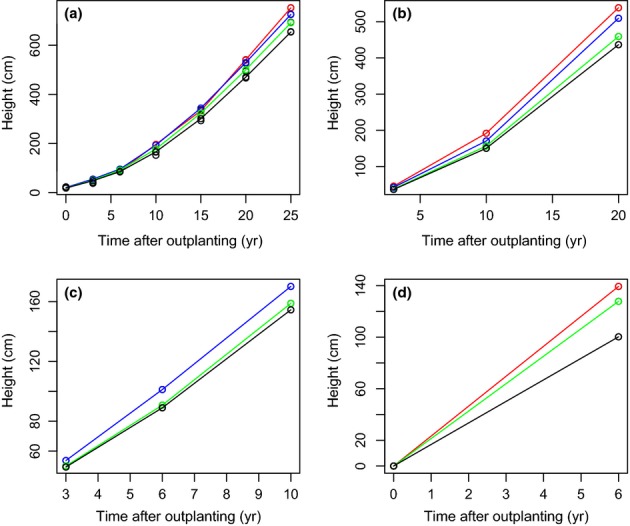
Differences in height among *Picea glauca*,* Picea engelmannii* , and their hybrids tested in sites of contrasting elevation. Intermediate-elevation tests sites are represented by (a) Quesnel and (b) East Kootenay; a high-elevation test site by (c)West Kootenay; and a low-elevation test site by (d) Fort Nelson. Red lines and dots indicate height measurements from *P. glauca*; blue, *P. glauca*-like hybrids; green, *P. engelmannii*-like hybrids; and black, *P. engelmannii*.

Significant differences in height were found among genotypic classes for all ages measured in the East Kootenay, Quesnel, and Fort Nelson test sites. In the West Kootenay hybrid habitat test sites, height differences were significant at ages 3 and 6 yr, but not at age 10 yr (Table2, Fig.2). Differences among genotypic classes were not significant for height at any ages assessed for the high-elevation Engelmann habitat West Kootenay site. introgress and NewHybrids showed similar results, with the exception of height at age 6 yr in the West Kootenay SPZ hybrid test sites (Table S1).

**Table 2:** Differences among genotypic classes for phenotypic traits based on ANOVA results

Traits	*P. engelmannii* environment		Hybrid environment						*P. glauca* environment	
	West Kootenay		East Kootenay		West Kootenay		Quesnel		Fort Nelson	
	*F*-value (df = 2)	*P*-value	*F*-value (df = 3)	*P*-value	*F*-value (df = 2)	*P*-value	*F*-value (df = 3)	*P*-value	*F*-value (df = 2)	*P*-value
Height age 3 yr	1.89	0.1697	**22.56**	**< 0.0001**	**3.96**	**0.01**	**20.73**	**< 0.0001**	–	–
Height age 6 yr	1.34	0.2464	–	–	**3.17**	**0.04**	**23.68**	**< 0.0001**	**81.35**	**< 0.0001**
Height age 10 yr	1.43	0.2326	**13.71**	**< 0.0001**	1.9	0.15	**46.56**	**< 0.0001**	–	–
Height age 20 yr	–	–	**20.27**	**< 0.0001**	–	–	**32.05**	**< 0.0001**	–	–
Frost injury −25°C	0.01	0.9332	1.23	0.2982	–	–	0.19	0.829	–	–
Bud burst	0.66	0.5182	1.63	0.1983	–	–	**3.28**	**0.0223**	–	–
Bud set	–	–	**25.09**	**< 0.0001**	–	–	–	–	–	–

Genotypic classes are classified as pure *Picea engelmannii*, pure *Picea glauca*, advanced generation hybrids (Fn) and first-generation hybrids (F1), based on the NewHybrids assignment. In the Fort Nelson test site, pure species and hybrids are differentiated by elevation and geographical location of populations of origin. All genotypic classes are present at East Kootenay, Quesnel and Fort Nelson sites; at West Kootenay there is no pure *P. glauca*. Traits showing significant differences are indicated in bold text. Results of the introgress assignment can be found in Supporting Information Table S1. Number of individuals per genotypic class, means and standard errors for individual traits are provided in Tables S2 and S3.

*Picea glauca* and *P. glauca*-like hybrids grew faster on average than *P. engelmannii* and *P. engelmannii*-like hybrids in intermediate- and low-elevation environments (Fig.2, Tables S2 and S3). In some hybrid sites, however, *P. glauca* and *P. glauca*-like hybrids did not show greater height growth than *P. engelmannii* until 10–15 yr of age. *Picea engelmannii* and *P*. *engelmannii*-like hybrids may, in fact, show faster growth at early seedling and sapling stages. In the *P. engelmannii* habitat of the WK high-elevation test site, there were no significant differences between the heights of *P*. *engelmannii* and hybrid individuals. There were no pure *P. glauca* individuals planted at this site.

Trees classified as first-generation hybrids (F1) by NewHybrids had extremely variable phenotypes (Table S3). For example, F1 hybrids grew taller on average than advanced generation hybrids and *P. engelmannii* in Quesnel, but showed the opposite trend in West Kootenay. In East Kootenay, F1s grew similarly to *P. engelmannii*, but then at age 20 yr surpassed *P. engelmannii* and reached sizes similar to those of the advanced generation hybrids.

**Figure 3:**
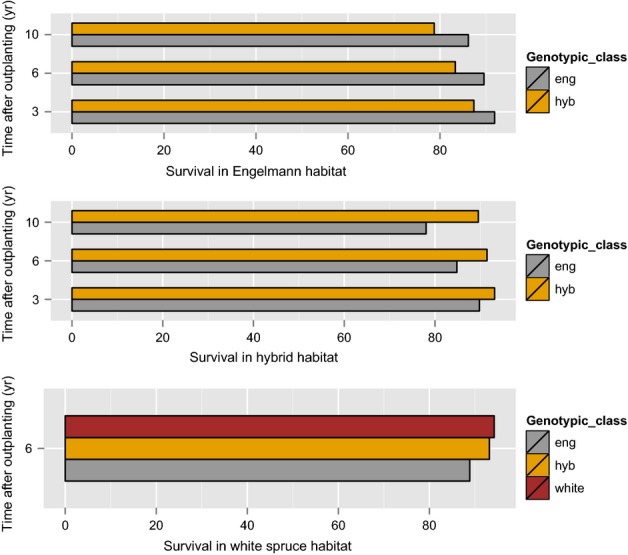
Results of the survival analysis for *Picea glauca*,* Picea engelmannii* and hybrids suggest that pure species and hybrids have better survival in their own habitat. Percentage survival was compared between hybrid and pure species in the high-elevation test site in Cortiana, West Kootenay (WK;* P. engelmannii* habitat); the low-elevation test site in Fort Nelson (*P. glauca* habitat); and across nine intermediate-elevation sites in East Kootenay (EK), Quesnel Lakes (QL) and WK (*P. glauca* × *P. engelmannii* hybrids habitat). Differences between pure species and hybrids are significant at all ages. Survival estimates for all tests sites can be found in Supporting Information Table S5.

Binomial tests indicated there were significant differences in survival at different ages among *P*. *glauca*,* P. engelmannii* and their hybrids (Table S4). On average across all hybrid habitat sites, hybrids had a higher survival rate than pure species in intermediate environments at all ages with the exception of age 20 yr (Fig.3, Table S5). At age 20 yr, the pattern is maintained in all the hybrid sites with the exception of Little Benson and Ketcham Creek (Table S5). *Picea engelmannii* had a higher survival rate than hybrids in the high-elevation site, and a lower survival rate than *P. glauca* and hybrids in the low-elevation Fort Nelson test site (Fig.3, Table S5).

Bud burst timing was negatively correlated with hybrid index in Quesnel (*R*^2^ = 0.28; *P *<* *0.0001) and East Kootenay (*R*^2^ = 0.103; *P *=* *0.021), with *P. engelmannii* and *P. engelmannii-*like hybrids having an earlier bud burst than *P. glauca* and *P. glauca*-like hybrids in the same environment. Bud burst phenology was also negatively correlated with elevation in Quesnel (*R*^2^ = 0.12; *P *=* *0.009) and in East Kootenay (*R*^2^ = 0.2557; *P *=* *0.0002). Bud burst timing was significantly different among *P. glauca*,* P. engelmannii* and their hybrids in East Kootenay and Quesnel but not in West Kootenay based on the introgress classification, but differed among NewHybrids genotypic classes only in Quesnel (Table2, Table S1).

Bud set timing, which was only recorded in the East Kootenay SPZ, was also negatively correlated with hybrid index (*R*^2^ = 0.126; *P *=* *0.0003), elevation (*R*^2^ = 0.231; *P *<* *0.0001) and latitude (*R*^2^ = 0.025; *P *=* *0.035) in East Kootenay. *Picea engelmannii* and *P. engelmannii*-like hybrids had earlier bud set timing than *P. glauca* and *P. glauca*-like hybrids. Although hybrids were generally more cold-hardy, on average, than pure species in intermediate environments, differences between hybrids and pure species were significant only in Quesnel (*F *=* *3.58; *P *=* *0.0297). Injury at −25°C was significantly but weakly correlated with elevation (*R*^2^ = 0.128; *P *=* *0.0072) in Quesnel, and with longitude (*R*^2^ = 0.108; *P *=* *0.019) in East Kootenay.

### Relationships between climate and hybrid index

The hybrid index showed strong clines corresponding to geographical and climatic gradients in temperature and precipitation (Table S7). The main climatic variables associated with variation in the hybrid index across all SPZs were precipitation as snow (PAS), mean annual precipitation (MAP) and the summer heat-moisture index (SHM). SHM is a measure of aridity calculated using mean warmest month temperature (MWMT) divided by adjusted mean summer precipitation (MSP/1000).

**Figure 4:**
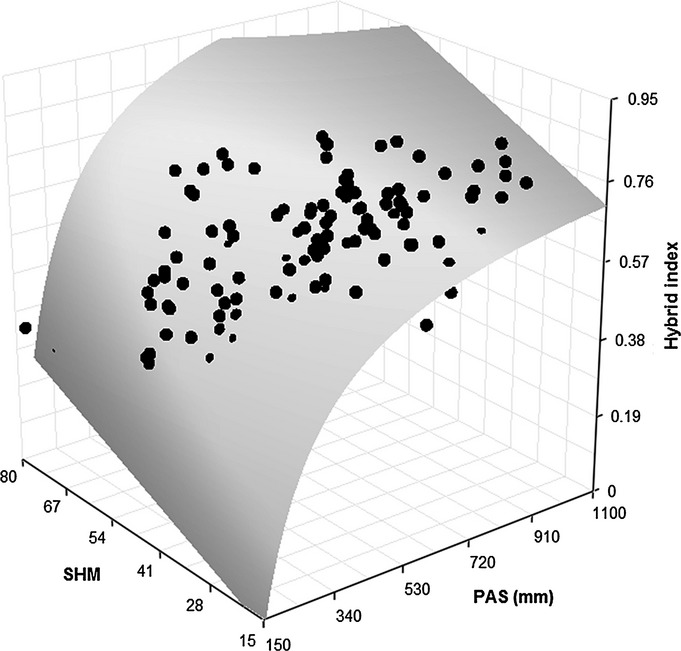
Observed (circles) and predicted (surface) spruce hybrid index against precipitation as snow (PAS) and summer heat-moisture index (SHM). *Picea glauca* × *Picea engelmannii* predicted hybrid index was generated based on the multiple linear relationships between hybrid index and climate variables shown in Table S7.

A stepwise multivariate regression of the hybrid index on climate variables found the best fit for a model that included 1/PAS and SHM. This model explained 54% of the total variation in the hybrid index (Table S6). The hybrid index was positively correlated with PAS (*R*^2^ = 0.445; *P *<* *0.0001; Fig.4) and SHM added modestly to model fit (partial *R*^2^ = 0.077; *P *<* *0.0001). Univariate regressions for SHM accounted for relatively little of the total variation (*R*^2^ = 0.108; *P *<* *0.0001). SHM is negatively correlated with the hybrid index in the univariate model, but positively when combined with PAS.

**Figure 5:**
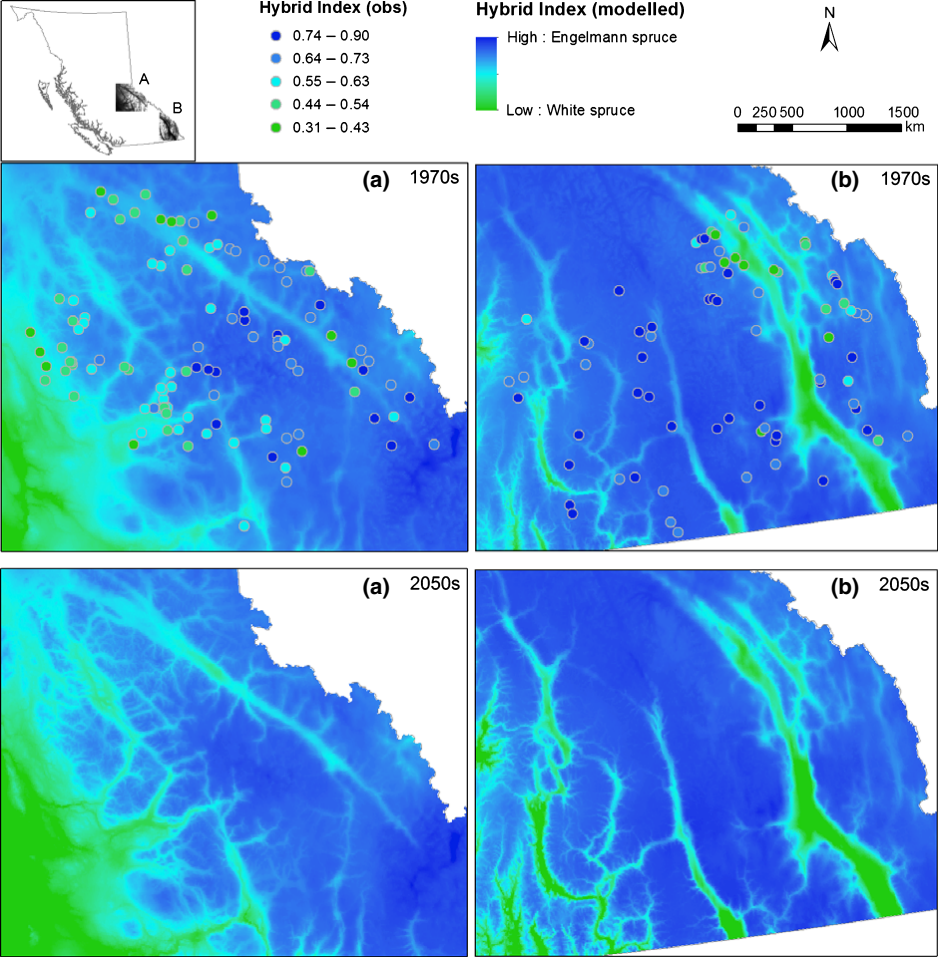
Observed (circles) and predicted (surface) hybrid index in the two sampling areas. The predicted hybrid index for the 2050s (lower two maps) was based on projected climate for this period by the Canadian third-generation coupled global climate model CGCM3 A2 run4. Blue colour indicates *Picea engelmannii* and green colour, *Picea glauca*.

The relatively strong relationship between the hybrid index and the two most explanatory climate variables, PAS and SHM, enabled the prediction of hybrid index values over all SPZs combined for the reference period 1961–1990. Maps of predicted current distribution of the hybrid index showed good matches with observed ones (Fig.5). However, the matches were better in Mount Robson and Quesnel SPZ than in the East Kootenay and West Kootenay SPZs. Optimal hybrid index values for projected climates in 2050s were expected to decline to favour *P. glauca* as a result of the forecasted decrease in PAS. However, the decline in optimal hybrid index was predicted to be more rapid in areas with low PAS, while the change was slight at high PAS because of the nonlinear relationship between the two climate variables and the hybrid index (Fig.4).

### Effects of artificial selection on hybrid index

**Figure 6:**
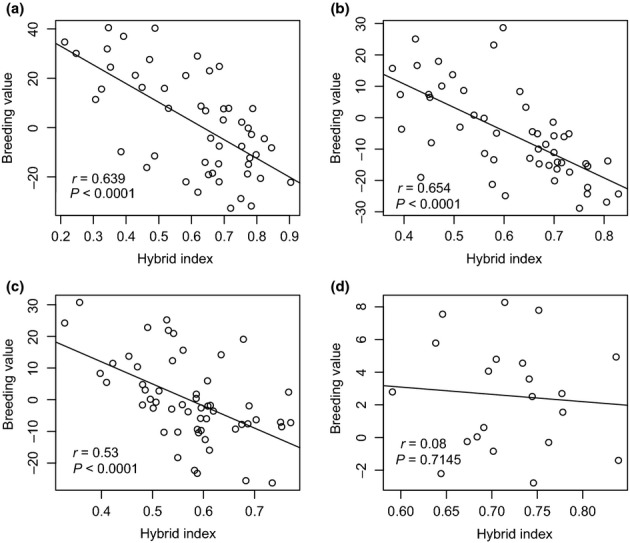
Breeding values versus family mean hybrid index for spruce in four seed planning zones (SPZs) represented in this study: (a) East Kootenay (EK), (b) Mount Robson (MR), (c) Quesnel (QL) and (d) West Kootenay (WK). Circles represent families with the potential for breeding based on stem volume (positive and high breeding values) or with no potential for breeding (negative or low breeding values). Individuals with a lower hybrid index are more *Picea glauca*-like, while those with higher hybrid indices are more *Picea engelmannii*-like.

Breeding values were negatively correlated with the hybrid index in three of the four SPZs studied. Pearson correlation coefficients (*r*) were −0.64 for East Kootenay, −0.65 for Mount Robson, and −0.53 for Quesnel (Fig.6). In these three SPZs, families with higher breeding potential were mainly composed of *P. glauca*-like hybrids because pure *P. glauca* tends to grow faster than *P. engelmannii*. It is important to note, however, that there is a fairly wide range of BVs for any given hybrid index value, and that these estimates were based on different progeny tests in each zone. In the West Kootenay SPZ, correlations between hybrid index and breeding values were not significant, probably as a consequence of the small range in hybrid index (between 0.6 to 1), where all individuals were either pure *P. engelmannii* or advanced generation *P*. *engelmannii*-like hybrids, with a correspondingly small range in breeding values (−2 to 8).

## Discussion

### Fitness analysis

Boreal woody species like *P. glauca* have an outstanding capacity to survive deep winter freezing temperatures. Their relative fitness depends on the ability of individuals to maximize their growth to be able to compete for light and yet avoid frost and drought injury by synchronizing growth phenology with the local climate (Howe *et al*., 2003). Individuals with a higher fitness will be those that grow taller and yet stop growing (i.e., set a terminal bud, enter dormancy, and initiate cold acclimation) before the first frost in that environment. In subalpine environments, species like *P. engelmannii* need the ability to survive a long winter beneath a deep and persistent snowpack, and accomplish growth in a short available window in cool temperatures and cold soil. Thus, while both species inhabit cold environments, the climatic selection pressures in these environments differ substantially.

Hybrids have a combination of *P. engelmannii* and *P. glauca* genomes; therefore, they have inherited a combination of genes that may provide them with an adaptive advantage in intermediate environments. Hybrids will generally grow taller than pure *P. engelmannii,* but will set bud earlier than *P. glauca*. This combination of faster growth and earlier bud set gives the hybrids an environment-specific survival advantage during seedling stages. This probably explains the higher hybrid survival rates at early ages found in this study. These conditions hold in intermediate environments (hybrid habitats); however, the story is different in pure species habitats.

In low-elevation environments (*P. glauca* habitats), hybrids cannot compete with faster growing *P. glauca* (Table2). In high-elevation environments (*P. engelmannii* habitats), hybrids probably cannot withstand the heavy snow loads or long, cold winters to which pure *P. engelmannii* is adapted. The minimum winter temperatures experienced are moderated by the snowpack, but persistence under the snow presents other challenges by shortening growing season length and exposing trees to pathogens. This could explain why hybrids showed lower survival rates than *P. engelmannii* in the high-elevation test site, and lower survival rates than *P. glauca* in the low-elevation test site.

Although it was already well known that *P. glauca* had faster growth than *P. engelmannii*, what was not previously known is that this trend in growth rate is not evident at all life history stages. This study indicates that *P. glauca* does not attain a size advantage until later in life, around age 10 yr. During the first years as seedlings and saplings, *P. glauca* sometimes grows less than hybrids or *P. engelmannii*. This growth lag at early ages may be related to a lower capacity to withstand heavy snow loads and shorter growing seasons than *P. engelmannii* and hybrids. Once a tree is well established, it will be less susceptible to frost injury and therefore more energy resources can be allocated to greater growth (Sakai & Larcher, 1987). Drought hardiness also plays a role in the relative performance and growth trajectories of these species and their hybrids*. Picea engelmannii* grew more slowly and suffered higher mortality at drier sites in provenance trials in East Kootenays (Xie *et al*., 1998).

Bud set timing is generally under strong divergent selection, exhibits strong local adaptation, and occurs as a genetically determined response to photoperiod, whereas bud burst is triggered by the accumulation of a genetically determined heat sum after the exposure to chilling temperatures and shows high phenotypic plasticity and weaker local adaptation (Howe *et al*., 2003). While these traits are determined by different environmental cues, the timing of bud burst followed the same clinal pattern as bud set in this study, as *P. engelmannii* and *P. engelmannii*-like hybrids both burst and set buds earlier than *P. glauca* and *P*. *glauca*-like hybrids.

### Climate analysis

Our climate analyses suggest that the hybrid zone is primarily maintained by local adaptation to winter temperatures, winter precipitation, and summer aridity, with the most important variables being PAS and SHM. PAS is based on monthly precipitation and monthly mean temperature (Wang *et al*., 2006). A larger value of PAS, or a deeper snowpack, can reflect a larger amount of winter precipitation, cooler temperatures, or longer winters. PAS explained 46% of the total variation in hybrid index, and it is likely to be the main factor limiting *P. glauca* survival in *P. engelmannii* habitats (high-elevation environments). The complex interaction between summer moisture (SHM) and early cold events has a strong effect on fitness and certainly on species distribution.

### Exogenous selection as mechanism of maintenance of the hybrid zone

Our results indicate that the *P. glauca* × *P. engelmannii* hybrid zone is maintained by elevational climatic selection gradients resulting from environmental heterogeneity (exogenous selection), where hybrids are fitter than parental species in intermediate habitats. Adaptive introgression from *P. engelmannii* to *P. glauca* has produced hybrids with increased fall cold tolerance as a result of an earlier bud set. At the same time, adaptive introgression from *P. glauca* to *P. engelmannii* has resulted in hybrids with faster growth rates. This combination of faster growth and increased cold tolerance has given the hybrids an adaptive advantage over parental species (pure *P. glauca* and pure *P. engelmannii*) in intermediate environments. This type of adaptive introgression has been reported in other plant species. Introgression from the flood-tolerant *Iris fulva* into the dry-adapted *Iris brevicaulis* has increase flooding tolerance in *I. fulva* backcrosses (Martin *et al*., 2005, 2006). Also, adaptive introgression from *Helianthus debilis* into *Helianthus annuus* has increased hervibory resistance (Whitney *et al*., 2006) and abiotic tolerance in several traits (Whitney *et al*., 2010).

Hybrids usually appear fitter than parentals in intermediate environments, and less fit than both parentals in their respective native environments. This suggests that the *P. glauca* × *P. engelmannii* zone is maintained according to the bounded hybrid superiority model (Moore, 1977). The most well-characterized example of bounded hybrid superiority is the hybrid zone formed between subspecies of the long-lived, wind-dispersed big sagebrush, *Artemisia tridentata* (Wang *et al*., 1997; Miglia *et al*., 2005). The *Artemisia* hybrid zone occurs along a much narrower elevational gradient than that in this study. *Artemisia* reciprocal transplant experiments have shown that hybrids are fitter than parentals in intermediate environments. Strong elevational and habitat differentiation among pure species and hybrids habitats have also been found in *Aquilegia* (Hodges & Arnold, 1994), *Ipomopsis* (Campbell & Waser, 2007) and *Carex* (Choler *et al*., 2004).

The most likely explanation for the low numbers of *P. glauca* backcrosses is the higher local and regional population density of *P. engelmannii* than *P. glauca*. Our previous molecular marker studies have found that pure *P. engelmannii* appears to be present at a higher frequency than *P. glauca* within the contact zone, assuming that the samples genotyped provide a proportional representation of the parental species and their hybrids (A. R. De La Torre & S. Aitken, unpublished data). If *P. glauca* individuals were more common in this area, then there would be more opportunities for the hybrids to backcross with them. The low numbers of *P. glauca* backcrosses may also be explained by endogenous selection, for example, as a result of Dobzhansky–Muller genetic incompatibilities, which we cannot test using our data but cannot be completely ruled out. It may be that introgression from *P. glauca* into *P. engelmannii*, besides generating fit hybrids (hybrids with higher growth potential), also generates some unfavourable epistatic combinations that lead to unfit hybrids. Artificial crosses for at least two generations would be required to draw definitive conclusions about this.

Given the complexity of this hybrid zone, it is difficult to differentiate between F2 hybrids and backcrosses. Therefore, they were included in the same hybrid category in the NewHybrids analysis. This lack of differentiation between hybrid classes, however, did not have a big impact, as only a small number of F1 hybrids were identified. This is not surprising, considering that the physical distance between parental habitats is not that great relative to pollen dispersal capabilities (Kremer *et al*., 2012), and that the hybrid zone is not of recent origin (Haselhorst & Buerkle, 2013). Even if F1s have low fitness, on average, they may mate with nonhybrid individuals to form early backcrosses that will then mate again with pure species to form advanced generation hybrids. This process would allow the transfer of adaptations from one species to the other (Arnold *et al*., 2012). In our study, apparent F1 hybrids showed high variability in the traits studied and did not show, on average, increased hybrid fitness. Advanced generation hybrids, however, were the most abundant genotypic class in the zone, showing, on average, higher hybrid fitness than F1s. These differences in fitness between hybrid classes are consistent with previous reports in natural hybrid zones (Arnold *et al*., 2012, but see Milne *et al*., 2003).

In contrast to artificial hybrid zones, in which only one or two generations are tested, individuals in this study comprised a vast array of recombinant hybrid genotypes, formed as a result of several to many generations of intercrossing (Rieseberg *et al*., 2000). Long-term phenotypic data (collected over a 20-yr period) also provided a rare opportunity to study the adaptation of these long-lived woody species. However, because populations were highly admixed, this study failed to elucidate the processes that occurred early in hybrid zone formation, as is expected with studies using ancient natural hybrid zones, and some remaining questions about fitness of early-generation hybrids will require multiple generations of artificial crosses to answer (Carney *et al*., 2000; Kirk *et al*., 2005).

### Effects of tree breeding and climate change on the hybrid zone

*Picea glauca* and *P. engelmannii* are economically important species in Canada, with tens of millions of trees planted annually for reforestation. In British Columbia, the complex of *P. glauca*,* P. engelmannii* and their hybrids is managed as a single ‘interior spruce’ species group within SPZs, based on geography and similarities in adaptive traits from provenance trials, and within seed planning units (SPUs), based on elevational bands within SPZs (Xie *et al*., 1998). SPZ classification is used for managing reforestation using seed collected from natural stands, with latitudinal, longitudinal and elevational limits on transfer distances from the collection site to the reforestation site. SPUs are used for managing reforestation with seed orchard lots from selective breeding programmes. Although this classification captures and manages most of the genetic variation at the macro-scale, it does not reflect the finer scale picture. Detailed knowledge of population structure and differences in adaptive traits among *P. glauca*,* P. engelmannii* and their hybrids fills this gap, and also provides key information for the basis of genetic gains from artificial selection for volume growth within this hybrid zone.

By 2050, the interior of British Columbia is expected to get warmer and drier, with a projected increase in mean annual temperature of 2–3°C according to projections of multiple General Circulation Models and glasshouse gas emissions scenarios (Mbogga *et al*., 2009). As a consequence of a predicted decrease in PAS, new climates should favour hybrids with higher *P. glauca* ancestry (hybrid index closer to 0) over those with more *P. engelmannii* ancestry (hybrid index closer to 1). However, as a consequence of the nonlinear relationship between hybrid index and PAS (Fig.4), and the moderate correlation between PAS and elevation (*R*^2^ = 0.416; *P *<* *0.0001), this trend favouring *P. glauca* will be most evident at low and intermediate elevations. At high elevations (elevation > 1700 m and PAS > 700), the hybrid index is not expected to change much. However, because of the uncertainty of future climates, accurate projections for these changes will, at least, require the use of multiple climate change scenarios and even a more sophisticated model to better capture this relationship in future studies.

Future climatic conditions will affect both natural and managed populations, but there is more opportunity with managed forests to shift genotypes to those better adapted to new conditions through practices such as climate-based seed transfer and assisted gene flow (Aitken *et al*., 2008; Aitken & Whitlock, 2013). However, our data suggest that managed populations may in fact be somewhat pre-adapted to climate change compared with natural populations. Our results indicate that artificial selection for breeding is currently selecting individuals that have more *P. glauca* ancestry because of their faster growth compared with *P. engelmannii*-like hybrids and pure *P. engelmannii*. This asymmetry towards *P. glauca* in managed populations may confer an adaptive advantage over natural populations in future warmer and drier climates, especially at low and intermediate elevations. This pattern of artificial selection in breeding programmes may counter the historical effects of natural selection on populations in the hybrid zone. Other studies using neutral and adaptive molecular markers in this hybrid zone have identified a strong asymmetry in introgression, with *P. engelmannii*-like hybrids much more abundant than the *P. glauca*-like hybrids (Haselhorst & Buerkle, 2013; A. R. De La Torre & S. Aitken, unpublished data). *Picea engelmannii*-like hybrids are adapted to shorter, cooler growing seasons and are slower growing than *P. glauca*-like hybrids; therefore they may be poorly adapted to new, warmer climates in British Columbia. However, the high levels of genetic variation found at the contact zone (intermediate elevations and intermediate latitudes) may increase the ability of hybrid populations to adapt to new climates in comparison with single species populations (Aitken *et al*., 2008).

This study represents a significant step forward in our understanding of the evolutionary dynamics that maintain the *P. glauca* × *P. engelmannii* hybrid zone and contributes valuable information for breeding and management of the species under climate change. Our ongoing research seeks to identify the genes responsible for adaptation to climate investigation of allele–environment associations and association mapping of genes underlying climate-related phenotypic traits.

## Supporting Information

Table S1 Differences among genotypic classes for phenotypic traits based on introgress hybrid index estimates **Table S2** Least square means (lsmeans) and standard errors (stderr) for height measurements (cm) at different ages among genotypic classes tested in seed planning zones based on introgress hybrid index estimates **Table S3** Least square means and standard errors for height measurements (cm) at different ages among hybrid classes tested in seed planning zones based on NewHybrids assignment **Table S4** Binomial tests for differences in survival at different ages among Picea glauca, P. engelmannii and their hybrids **Table S5** Differences in survival between Picea engelmannii, P. glauca and hybrids at different ages for all sites and regions studied in field common garden experiments **Table S6** Results of stepwise regression of hybrid index on precipitation as snow (PAS) and summer heat-moisture index (SHM) **Table S7** Results of the univariate regressions of hybrid index on geographical and climate variables in each seed planning zone
