# Supplementary material for: Adaptation and exogenous selection in a Picea glauca × Picea engelmannii hybrid zone: implications for forest management under climate change
Source: New Phytol. 2013 Oct 8;201(2):687–99. doi: 10.1111/nph.12540 (PMC4285121; doi:10.1111/nph.12540)
Supplement: Table S1 — Differences among genotypic classes for phenotypic traits based on introgress hybrid index estimates Table S2 Least square means (lsmeans) and standard errors (stderr) for height measurements (cm) at different ages among genotypic classes tested in seed planning zones based on introgress hybrid index estimates Table S3 Least square means and standard errors for height measurements (cm) at different ages among hybrid classes tested in seed planning zones based on NewHybrids assignment Table S4 Binomial tests for differences in survival at different ages among Picea glauca, P. engelmannii and their hybrids Table S5 Differences in survival between Picea engelmannii, P. glauca and hybrids at different ages for all sites and regions studied in field common garden experiments Table S6 Results of stepwise regression of hybrid index on precipitation as snow (PAS) and summer heat-moisture index (SHM) Table S7 Results of the univariate regressions of hybrid index on geographical and climate variables in each seed planning zone [file nph0201-0687-sd1.doc]

**Supporting Information**

**Table S1** Differences among genotypic classes for phenotypic traits based on *INTROGRESS* hybrid index estimates

|  | Hybrid environment | | | | | | *P. engelmannii* environment | |
| --- | --- | --- | --- | --- | --- | --- | --- | --- |
|  | East Kootenay | | West Kootenay | | Quesnel | | West Kootenay | |
| Traits | F-value | P-value | F-value | P-value | F-value | P-value | F-value | P-value |
|  | (df=3) |  | (df=2) |  | (df=3) |  | (df=2) |  |
| Height age 3 | **25.32** | **<.0001** | **4.05** | **0.0176** | **9.29** | **<.0001** | 0.88 | 0.4149 |
| Height age 6 | -- | -- | 1.49 | 0.2259 | **15.18** | **<.0001** | 2.03 | 0.1313 |
| Height age 10 | **14.45** | **<.0001** | 0.92 | 0.3968 | **24.7** | **<.0001** | 1.54 | 0.2152 |
| Height age 20 | **22.66** | **<.0001** | -- | -- | **21.74** | **<.0001** | -- | -- |
| Frost injury -25°C | 1.38 | 0.2477 | -- | -- | **3.58** | **0.0297** | 0.01 | 0.9332 |
| Bud Burst | **8.2** | **<.0001** | -- | -- | **3.74** | **0.006** | 0.56 | 0.5735 |
| Bud set | **4.05** | **0.0204** | -- | -- | -- | -- | -- | -- |

Genotypic classes are stratified as pure *P. engelmannii*, pure *P. glauca*, *P. glauca*-like hybrids, and *P. engelmannii*-like hybrids. All genotypic classes are present at East Kootenay and Quesnel sites; at West Kootenay there is no pure *P. glauca*. Traits showing significant differences are indicated in bold text. Number of individuals per genotypic class, and means and standard errors for individual traits are provided in Supporting Information Tables S2 and S3.

**Table S2** Least square means (lsmeans) and standard errors (stderr) for height measurements (cm) at different ages among genotypic classes tested in Seed Planning Zones Quesnel (QL), East Kootenay (EK), West Kootenay (WK) and Fort Nelson (FN). Genotypic classes are classified as pure *Picea glauca*, *P. glauca*-like hybrids (*hyb-glauca*), *P. engelmannii*-like hybrids (*hyb-eng*) and *P. engelmannii* (*P. eng*); based on *INTROGRESS* hybrid index. In the Fort Nelson test site, pure species and hybrids are differentiated by elevation and geographic location.

|  |  |  |  | Years after outplanting | | | | |
| --- | --- | --- | --- | --- | --- | --- | --- | --- |
| Region | Genotypic class | N | Variable | 3 | 6 | 10 | 15 | 20 |
| QL | *P. glauca* | 8 | lsmeans | 37.54 | 86.81 | 165.05 | 333.30 | 540.17 |
|  |  |  | stderr | 2.01 | 5.38 | 10.80 | 20.23 | 29.12 |
|  | *hyb-glauca* | 491 | lsmeans | 41.62 | 93.84 | 177.91 | 337.69 | 526.63 |
|  |  |  | stderr | 0.26 | 0.70 | 1.43 | 2.69 | 3.88 |
|  | *hyb-eng* | 1134 | lsmeans | 40.35 | 89.22 | 166.15 | 316.34 | 494.79 |
|  |  |  | stderr | 0.17 | 0.46 | 0.93 | 1.75 | 2.53 |
|  | *P. eng* | 79 | lsmeans | 38.82 | 84.03 | 151.94 | 292.67 | 466.73 |
|  |  |  | stderr | 0.62 | 1.66 | 3.36 | 6.31 | 9.10 |
| EK | *P. glauca* | 48 | lsmeans | 45.59 | -- | 191.79 | -- | 538.78 |
|  |  |  | stderr | 1.82 | -- | 8.46 | -- | 21.45 |
|  | *hyb-glauca* | 540 | lsmeans | 42.43 | -- | 170.48 | -- | 509.73 |
|  |  |  | stderr | 0.56 | -- | 2.64 | -- | 6.78 |
|  | *hyb-eng* | 1260 | lsmeans | 37.63 | -- | 156.60 | -- | 459.32 |
|  |  |  | stderr | 0.37 | -- | 1.73 | -- | 4.41 |
|  | *P. eng* | 396 | lsmeans | 36.50 | -- | 150.30 | -- | 436.64 |
|  |  |  | stderr | 0.67 | -- | 3.11 | -- | 7.95 |

**Table S2** (Continued) Least square means (lsmeans) and standard errors (stderr) for height measurements (cm) at different ages among genotypic classes tested in Seed Planning Zones Quesnel (QL), East Kootenay (EK), West Kootenay (WK) and Fort Nelson (FN). Genotypic classes are classified as pure *Picea glauca*, *P. glauca*-like hybrids (*hyb-glauca*), *P. engelmannii*-like hybrids (*hyb-eng*) and *P. engelmannii* (*P. eng*); based on *INTROGRESS* hybrid index. In the Fort Nelson test site, pure species and hybrids are differentiated by elevation and geographic location.

|  |  |  |  | Years after outplanting | | |
| --- | --- | --- | --- | --- | --- | --- |
| Region | Hybrid class | N | Variable | 3 | 6 | 10 |
|  | *hyb-glauca* | 19 | lsmeans | 58.91 | 104.48 | 169.73 |
| WK |  |  | stderr | 4.30 | 10.22 | 24.07 |
| Hall/Duhamel | *hyb-eng* | 1555 | lsmeans | 61.31 | 106.83 | 155.44 |
|  |  |  | stderr | 0.36 | 0.74 | 1.43 |
|  | *P. eng* | 575 | lsmeans | 63.19 | 109.27 | 158.93 |
|  |  |  | stderr | 0.58 | 1.23 | 2.44 |
| WK Cortiana | *hyb-glauca* | 10 | lsmeans | 53.70 | 101.20 | 170.20 |
|  |  |  | stderr | 3.69 | 6.60 | 12.72 |
|  | *hyb-eng* | 766 | lsmeans | 50.01 | 90.75 | 158.88 |
|  |  |  | stderr | 0.45 | 0.82 | 1.59 |
|  | *P. eng* | 294 | lsmeans | 49.32 | 88.97 | 154.54 |
|  |  |  | stderr | 0.72 | 1.30 | 2.53 |
| Fort Nelson | *P. glauca* | 213 | lsmeans | -- | 139.39 | -- |
|  |  |  | stderr | -- | 2.49 | -- |
|  | *all hybrids* | 2680 | lsmeans | -- | 127.84 | -- |
|  |  |  | stderr | -- | 0.67 | -- |
|  | *P. eng* | 224 | lsmeans | -- | 100.30 | -- |
|  |  |  | stderr | -- | 1.96 | -- |

**Table S3** Least square means and standard errors for height measurements (cm) at different ages among hybrid classes tested in Seed Planning Zones Quesnel (QL), East Kootenay (EK) and West Kootenay (WK). Genotypic classes are classified as pure *Picea engelmannii (P. eng)*, F1 hybrids, advanced generation hybrids (Fn) and pure *P. glauca*; based on *NewHybrids* assignment.

|  |  |  |  | Years after outplanting | | | | |
| --- | --- | --- | --- | --- | --- | --- | --- | --- |
| Region | Hybrid class | N | Variable | 3 | 6 | 10 | 15 | 20 |
| QL | *P. eng* | 347 | lsmean | 39.00 | 85.68 | 155.50 | 297.17 | 472.19 |
|  |  |  | stderr | 0.30 | 0.81 | 1.64 | 3.08 | 4.45 |
|  | F1 hybrids | 32 | lsmean | 43.22 | 98.76 | 190.07 | 357.54 | 545.23 |
|  |  |  | stderr | 1.03 | 2.79 | 5.64 | 10.64 | 15.46 |
|  | Fn hybrids | 1333 | lsmean | 41.00 | 91.31 | 171.87 | 326.99 | 509.93 |
|  |  |  | stderr | 0.16 | 0.43 | 0.86 | 1.63 | 2.35 |
| WK Hall/ Duhamel | *P. eng* | 1814 | lsmeans | 62.08 | 107.76 | 157.16 | -- | -- |
|  |  |  | stderr | 0.33 | 0.69 | 1.35 | -- | -- |
|  | F1 hybrids | 17 | lsmeans | 54.74 | 89.90 | 135.26 | -- | -- |
|  |  |  | stderr | 3.57 | 7.26 | 13.33 | -- | -- |
|  | Fn hybrids | 318 | lsmeans | 60.40 | 106.57 | 153.39 | -- | -- |
|  |  |  | stderr | 0.77 | 1.59 | 3.06 | -- | -- |
| EK | *P. eng* | 1164 | lsmeans | 36.62 | -- | 151.83 | -- | 444.03 |
|  |  |  | stderr | 0.39 | -- | 1.81 | -- | 4.64 |
|  | F1 hybrids | 24 | lsmeans | 37.74 | -- | 151.43 | -- | 494.35 |
|  |  |  | stderr | 3.44 | -- | 15.95 | -- | 40.64 |
|  | Fn hybrids | 1020 | lsmeans | 40.86 | -- | 167.43 | -- | 494.61 |
|  |  |  | stderr | 0.42 | -- | 1.92 | -- | 4.95 |
|  | *P. glauca* | 36 | lsmeans | 45.77 | -- | 182.79 | -- | 521.13 |
|  |  |  | stderr | 2.06 | -- | 9.56 | -- | 24.36 |

**Table S4** Binomial tests for differences in survival at different ages among *Picea glauca, P. engelmannii* and their hybrids.

|  | Hybrid environment | | | | | | *P. engelmannii* environment | |
| --- | --- | --- | --- | --- | --- | --- | --- | --- |
|  | East Kootenay | | West Kootenay | | Quesnel | | West Kootenay | |
| Traits | Z-value | P-value | Z-value | P-value | Z-value | P-value | Z-value | P-value |
| Survival age 3 | 3.538 | 0.0004 | 2.069 | 0.0385 | 2.210 | 0.0271 | -2.166 | 0.0303 |
| Survival age 6 | -- | -- | 2.575 | 0.005 | -5.338 | <0.0001 | -2.680 | 0.0037 |
| Survival age 10 | 3.618 | 0.0003 | 2.454 | 0.0141 | 2.450 | 0.0143 | -2.836 | 0.0046 |
| Survival age 20 | 2.884 | 0.0039 | -- | -- | 2.403 | 0.0162 | -- | -- |

**Table S5** Differences in survival between *Picea engelmannii (P.eng), P. glauca* and hybrids at different ages for all sites and regions studied in field common garden experiments. Significant differences are indicated in bold text (P<0.05). N indicates the number of samples included in the study.

| Genotypic class | Region | Site | N | Age 3 | Age 6 | Age 10 | Age 20 |
| --- | --- | --- | --- | --- | --- | --- | --- |
| *P. engelmannii habitat test site* | | |  |  |  |  |  |
| *P. eng* | W. Kootenay | Cortiana Ck | 1031 | **91.85** | **89.55** | **86.16** | -- |
| *hybrid* | W. Kootenay | Cortiana Ck | 174 | **87.36** | **83.33** | **78.74** | -- |
| *Hybrid habitat test sites* | | |  |  |  |  |  |
| *P. eng* | E. Kootenay | Bloom Ck | 194 | **89.17** | -- | **88.65** | 88.65 |
| *hybrid* | E. Kootenay | Bloom Ck | 174 | **95.97** | -- | **93.67** | 92.52 |
| *P. eng* | E. Kootenay | Perry Ck | 286 | **88.46** | -- | **87.41** | 86.01 |
| *hybrid* | E. Kootenay | Perry Ck | 247 | **93.11** | -- | **91.49** | 87.44 |
| *P. eng* | E. Kootenay | Red Rock | 538 | 93.68 | -- | **91.26** | 90.33 |
| *hybrid* | E. Kootenay | Red Rock | 422 | 95.26 | -- | **94.55** | 94.31 |
| *P. eng* | W. Kootenay | Hall Ck | 895 | **80** | **70** | **49** | -- |
| *hybrid* | W. Kootenay | Hall Ck | 155 | **85** | **78** | **58** | -- |
| *P. eng* | W. Kootenay | Duhamel Ck | 919 | 93.47 | 88.46 | 76.27 | -- |
| *hybrid* | W. Kootenay | Duhamel Ck | 180 | 95 | 90.55 | 78.88 | -- |
| *P. eng* | Quesnel | Little Benson | 347 | 95.1 | 94.81 | **93.08** | 91.93 |
| *hybrid* | Quesnel | Little Benson | 1364 | 92.88 | 92.08 | **90.46** | 89.73 |
| *P. eng* | Quesnel | Camp Ck | 367 | 95.45 | 99.18 | 98.36 | 97.27 |
| *hybrid* | Quesnel | Camp Ck | 1342 | 98.8 | 98.58 | 98.13 | 97.39 |
| *P. eng* | Quesnel | Ketcham Ck | 367 | 89.64 | 88.01 | **86.37** | **85.55** |
| *hybrid* | Quesnel | Ketcham Ck | 1373 | 87.47 | 85.5 | **82.81** | **81.06** |
| *P. eng* | All hybrid sites | | 3913 | **89.8** | **84.81** | **78.03** | **91.21** |
| *hybrid* | All hybrid sites | | 5257 | **93.12** | **91.45** | **89.54** | **89.77** |
| *P. glauca habitat test site* | | |  |  |  |  |  |
| *P. eng* | Fort Nelson | Fort Nelson | 224 | -- | **88.88** | -- | -- |
| *hybrid* | Fort Nelson | Fort Nelson | 2680 | -- | **93.2** | -- | -- |
| *P. glauca* | Fort Nelson | Fort Nelson | 213 | -- | **94.24** | -- | -- |

**Table S6** Results of stepwise regression of hybrid index on precipitation as snow (PAS) and summer heat-moisture index (SHM)

| Independent variable | Parameter  estimate | Partial  R^2^ | Model  R^2^ | *P*-value |
| --- | --- | --- | --- | --- |
| Intercept | 0.742 |  |  |  |
| 1/PAS | -126.764 | 0.458 | 0.458 | <0.0001 |
| SHM | 0.00524 | 0.078 | 0.536 | <0.0001 |

**Table S7** Results of the univariate regressions of hybrid index on geographical and climate variables in each Seed Planning Zone. P-values were adjusted for multiple comparisons using Bonferroni correction.

|  | **Quesnel** | | **Mount Robson** | | **East Kootenay** | | **West Kootenay** | |
| --- | --- | --- | --- | --- | --- | --- | --- | --- |
| **Variable** | **R**^2^ | **P>F** | **R**^2^ | **P>F** | **R**^2^ | **P>F** | **R**^2^ | **P>F** |
| Elevation | 0.144 | <.0001 | 0.474 | <.0001 | 0.422 | <.0001 | 0.195 | 0.299 |
| Latitude | 0.037 | 1 | 0.345 | <.0001 | 0.11 | 0.4025 | 0.019 | 1 |
| Longitude | 0.257 | <.0001 | 0.319 | <.0001 | 0 | 1 | 0.008 | 1 |
| Mean annual temperature (MAT) | 0.19 | 0.0207 | 0.504 | <.0001 | 0.248 | 0.0046 | 0.125 | 1 |
| Mean warmest month temperature (MWMT) | 0.13 | 0.1541 | 0.32 | <.0001 | 0.226 | 0.0092 | 0.088 | 1 |
| Mean coldest month temperature (MCMT) | 0.178 | 0.0299 | 0.396 | <.0001 | 0.006 | 1 | 0.067 | 1 |
| Continentality (TD) | 0.049 | 1 | 0.013 | 1 | 0.375 | <.0001 | 0.034 | 1 |
| Mean annual precipitation (MAP) | 0.197 | 0.0161 | 0.394 | <.0001 | 0.203 | 0.0207 | 0.015 | 1 |
| Mean summer precipitation (MSP) | 0.121 | 0.2116 | 0.169 | 0.0713 | 0.079 | 1 | 0 | 1 |
| Annual heat: moisture index (AHM) | 0.24 | 0.0023 | 0.539 | <.0001 | 0.242 | 0.0069 | 0.063 | 1 |
| Summer heat: moisture index (SHM) | 0.158 | 0.0621 | 0.284 | <.0001 | 0.121 | 0.2852 | 0.016 | 1 |
| Degree days below 0°C (D0) | 0.201 | 0.0138 | 0.542 | <.0001 | 0.134 | 0.1886 | 0.123 | 1 |
| Degree days above 5°C (D5) | 0.152 | 0.0736 | 0.366 | <.0001 | 0.308 | <.0001 | 0.118 | 1 |
| Degree days below 18°C (D18) | 0.187 | 0.023 | 0.508 | <.0001 | 0.246 | 0.0046 | 0.127 | 1 |
| Degree days above 18°C (DD18) | 0 | 1 | 0 | 1 | 0.065 | 1 | 0.064 | 1 |
| Number of frost-free days (NFFD) | 0.117 | 0.2415 | 0.451 | <.0001 | 0.182 | <.0001 | 0.091 | 1 |
| Julian date in which FFP begins (bFFP) | 0.12 | 0.2185 | 0.223 | 0.0115 | 0.105 | 0.4738 | 0.081 | 1 |
| Julian date in which FFP ends (eFFP) | 0.077 | 0.9361 | 0.27 | 0.0023 | 0.136 | 0.1794 | 0.05 | 1 |
| Frost-free period (FFP) | 0.106 | 0.3496 | 0.251 | 0.0046 | 0.123 | 0.2668 | 0.072 | 1 |
| Precipitation as snow (PAS) | 0.244 | 0.0023 | 0.509 | <.0001 | 0.254 | 0.0046 | 0.053 | 1 |
| Extreme minimum temperature over 30 years (EMT) | 0.029 | 1 | 0.513 | <.0001 | 0.04 | 1 | 0.054 | 1 |
| Hargreaves reference evaporation (Eref) | 0.205 | 0.0115 | 0.269 | 0.0023 | 0.249 | 0.0046 | 0.107 | 1 |
| Hargreaves climatic moisture deficit (CMD) | 0.188 | 0.0207 | 0.296 | <.0001 | 0.161 | 0.0828 | 0.046 | 1 |
